# Cellulose-Copper Oxide hybrid nanocomposites membranes for H_2_S gas detection at low temperatures

**DOI:** 10.1038/s41598-020-60069-4

**Published:** 2020-02-19

**Authors:** Waseem Hittini, Ayah F. Abu-Hani, N. Reddy, Saleh T. Mahmoud

**Affiliations:** 1 Department of Physics, UAE University, Al Ain, United Arab Emirates; 2 Department of Computer Engineering, German Jordanian University, German, Jordan

**Keywords:** Materials science, Sensors and biosensors

## Abstract

We report on novel, sensitive, selective and low-temperature hydrogen sulfide (H_2_S) gas sensors based on metal-oxide nanoparticles incorporated within polymeric matrix composites. The Copper-Oxide (CuO) nanoparticles were prepared by a colloid microwave-assisted hydrothermal method that enables precise control of nanoparticle size. The sodium carboxymethyl cellulose (CMC) powder with 5% glycerol ionic liquid (IL) was prepared and mixed with different concentrations of CuO NPs (2.5–7.5 wt.%) to produce flexible and semi-conductive polymeric matrix membranes. Each membrane was then sandwiched between a pair of electrodes to produce an H_2_S gas sensor. The temperature-dependent gas sensing characteristics of the prepared sensors were investigated over the temperature ranges from 40 °C to 80 °C. The sensors exhibited high sensitivity and reasonably fast responses to H_2_S gas at low working temperatures and at a low gas concentration of 15 ppm. Moreover, the sensors were highly selective to H_2_S gas, and they showed low humidity dependence, which indicates reliable functioning in humid atmospheres. This organic-inorganic hybrid-materials gas sensor is flexible, with good sensitivity and low power consumption has the potential to be used in harsh environments.

## Introduction

Hydrogen sulfide gas (H_2_S) is a major air pollutant that is produced in a large quantity from industrial fields such as petroleum and gas drilling and refining, sewage treatment, coke ovens, kraft paper mills, and landfills^1,2^. H_2_S gas is toxic gas with a malodor of rotten eggs. It can damage human respiratory and nerve systems, causing the public to lose consciousness with a possibility to die at minimal concentrations as low as a few hundreds of ppm^3–5^. Therefore, improving H_2_S sensors in terms of sensitivity, selectivity, response time, power consumption, and the cost is needed for environmental and safety concerns.

Different materials and methods have been reported in the literature for H_2_S gas detection, including electrochemical (solid electrolyte) sensors^5^, optical sensors^6,7^, piezoelectric sensors^8,9^, and oxide-semiconductor sensors^10,11^. However, most of these sensors developed using such methods suffer from high fabrication cost, high power consumption, poor stability, and malfunction in harsh environment^12,13^. Therefore, these sensors have been under continuous development to meet the growing demand of high-performance sensors. Metal-oxide semiconducting nanoparticles based sensors are the most promising materials for the H_2_S gas detection; they are cost-effective, easy to operate and fast in response with high sensitivity to the target gas^14,15^. Therefore, the development of new sensors that include polymer membranes and metal-oxide nanoparticles (organic-inorganic sensors) is expected to enhance the functionalities of such sensors; as they are flexible, easy to fabricate, and can be operated at low temperature with a low electrical power requirement^14–16^. Metal-oxide based sensors are flexible, easy to fabricate, and can be operated at low temperature with a low electrical power requirement^17–19^. Fabrication of electronic devices based on organic materials and inorganic nanomaterials has been intensively studied because they enable applications, such as transparent and flexible electronic devices, which are power saving, size compactable, and easily portable^18–20^.

Recent studies have incorporated metal-oxide nanoparticles in organic materials and tested their gas sensing properties. Metal-oxide-organic materials’ sensor showed enhanced sensing properties in terms of power saving, portability and size compactness^20^. Use of these hybrid materials would help in optimizing H_2_S sensors performance namely: selectivity, low power consumption, sensitivity and flexibility^16,21,22^.

The electrical conductivity of the organic-inorganic compounds can be controlled by doping the organic polymer with a suitable ionic liquid (IL) such as sorbitol, 1-methyl-3-n-decyl-imidazolium bromide, and glycerol^23–25^. In general, ILs serve as electrolytes and diffusion barriers, and they have low values of vapor pressure, wide potential window, low toxicity, and they are environmentally friendly. Due to their low-molecular-weight, when ILs are blended with polymers, the free volume of the material or the macro-molecular mobility of the polymer will increase, resulting in a decrease in the intermolecular forces, thus, the polymeric the network becomes less dense, and consequently the extensibility and flexibility of the membranes are improved^26^.

Among metal-oxides, copper oxide (CuO) is a highly sensitive material to H_2_S gas as reported in previous literature^21,26,27^. Thus, nanostructured CuO combination with a semiconducting polymer membrane is expected to exhibit enhanced sensitivity to H_2_S gas due to the larger surface to volume ratio of the nanostructured CuO, thus more reactive sites, and this would promote the chemical reactivity with the gas.

Sodium carboxymethyl cellulose (CMC) is an organic polymer with relatively low-cost, low density, available in nature, recyclable, and non-toxic material. Properties of cellulose have become highly important and have contributed to increasing the interest in this material^28^. CMC that has been widely utilized as supporting material or a reductant for the synthesis of gold, silver and platinum NPs^29–31^. Recently, few studies investigate the use of CMC composites as humidity^31^, hydrogen^32^ and Liquid petroleum gas (LPG) sensors^33^. To best of the authors’ knowledge, sensing properties of CMC composite towards H_2_S sensing was never tested before. Therefore, CMC was used in this work to fabricate H_2_S gas sensor based on CuO NPs and glycerol. Electrical properties, detection limit, selectivity and sensitivity of the prepared sensor was tested. Furthermore, the effect of the humidity on the sensor performance is investigated.

## Experimental

### Materials

Dimethylformamide (CH_3_)_2_NC(O)H (DMF) with a purity of 99.0% and copper(II) acetate monohydrate Cu(CH_3_COO)_2_·H_2_O with a purity of 99.8% were used for the preparation of copper nanoparticles. Sodium carboxymethyl cellulose (CMC) with average molecular weight (M_w_) 700000 was used for the preparation of CMC solution. All chemicals were purchased from Sigma Aldrich-USA. Glycerol was used as an ionic liquid and was purchased from Quartek Corp.-USA.

### Synthesis of CuO nanoparticles

For the synthesis of CuO nanoparticles, a colloid microwave-assisted hydrothermal process following a protocol that was described in^34^. An amount of 60 mg of Cu(CH_3_COO)_2_·H_2_O was dissolved in 50 ml of DMF under continuous stirring for 3 h at 25 °C. This solution was then aged for 24 h before exposing it to microwave radiations in a microwave (CEM, Discover-SP system, 909156, USA) at 45 °C with a power of 300 W and radiation frequency of 2455 MHz. The solution was kept under continuous stirring to complete the reaction and nucleation process. The working cycle of the microwave reactor was set as 30 s on and 15 s off repeatedly for 10 times.

### Preparation of CMC-CuO nanocomposite Membranes

**Figure 1:**
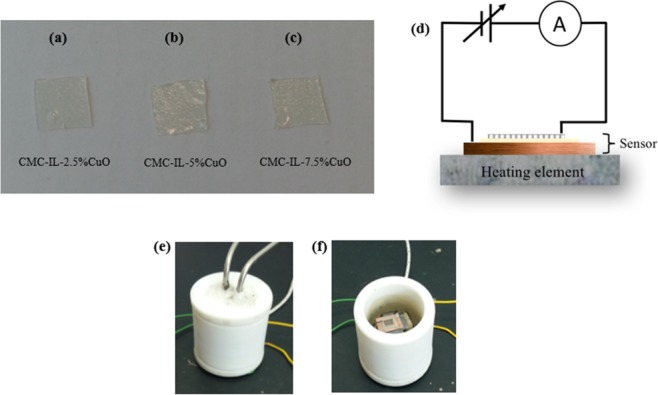
(**a**–**c**) Pictures of the prepared membranes with different CuO concentrations, (**b**) schematic diagram of the electrical measurement circuit (**e**) picture of the gas test chamber, (**f**) the sensor is shown inside the test chamber.

A 2 vol. % solution of CMC was first prepared by dissolving 2 g of sodium carboxymethyl cellulose in 100 ml of double-distilled water. The solution was kept under continuous stirring for 12 hours at room temperature. As a result, a clear homogeneous CMC solution was obtained. Next, different concentrations of the synthesized CuO nanoparticles (2.5 wt., 5 wt. and 7.5 wt.% of the polymer weight) were mixed with a selected quantity of the cellulose solution (CMC) along with 5 vol. % glycerol. Each solution was stirred for one hour. Then, the three samples were kept to dry at 50 °C in a vacuum oven for 24 hours. Finally, transparent membranes of around 200 µm thickness were obtained as shown in Fig. 1(a–c).

### Device fabrication

Each membrane was cut into a (1 × 1 cm^2^) piece, as shown in Fig. 1(a–c). Then, a membrane was encapsulated between a (1.5 × 1.5 cm^2^) copper sheet of 0.15 μm thickness that was used as a back contact, and a stainless steel grid (0.8 × 0.8 cm^2^) with a grid size of 250 × 250 µm^2^ was used as a top electrode. Herein, stainless steel was chosen for the top contact since it is anti-corrosive against H_2_S, and its work function is comparable to other metals such as gold. The three layers were fixed using a strong heat-proof scotch tape suitable adhesion and the fabricated sensor was then electrically connected as illustrated in Fig. 1(d).

### Characterization techniques

The morphology of the CuO nanoparticles was investigated using transmission electron microscopy (TEM), and scanning electron microscopy (SEM) equipped with an energy-dispersive X-ray spectroscopy (EDS) apparatus that enables the evaluation of the elemental analysis of the CuO nanoparticles. The X-ray diffraction (XRD) of the CuO nanoparticles was performed using a Shimadzu 6100 x-ray diffractometer with Cu-Kα radiation (λ = 1.5406 Å). The particle size analysis of the CuO nanoparticles was also evaluated using a zeta-sizer (Malvern Instruments, Model ZEN360, England).

The electrical characteristics and gas sensing measurements of the sensors were performed using a Keithley Instruments (KI 236) source measurement unit while the sensor was placed inside a temperature-controlled chamber (Fig. 1(e,f)). The target gas was introduced into a gas flowmeter, and the flow rates were controlled using a Bronkhorst mass flow controller. Then, H_2_S gas was added in controlled concentrations, and it was diluted with air inside a gas mixer and injected into the chamber with controlled flow rates. The gas test chamber was placed inside a fume hood at 25 °C and relative humidity of 45% under atmospheric pressure. The relative humidity inside the test chamber was 0% (dry gas) unless otherwise is stated. Based on the current-voltage (I–V) characteristics of the sensor a constant voltage was applied across the sensor electrodes, and the electrical current signal was measured as a function of time and gas concentrations.

## Results and Discussion

### Morphology and structural characteristics of nanoparticles

**Figure 2:**
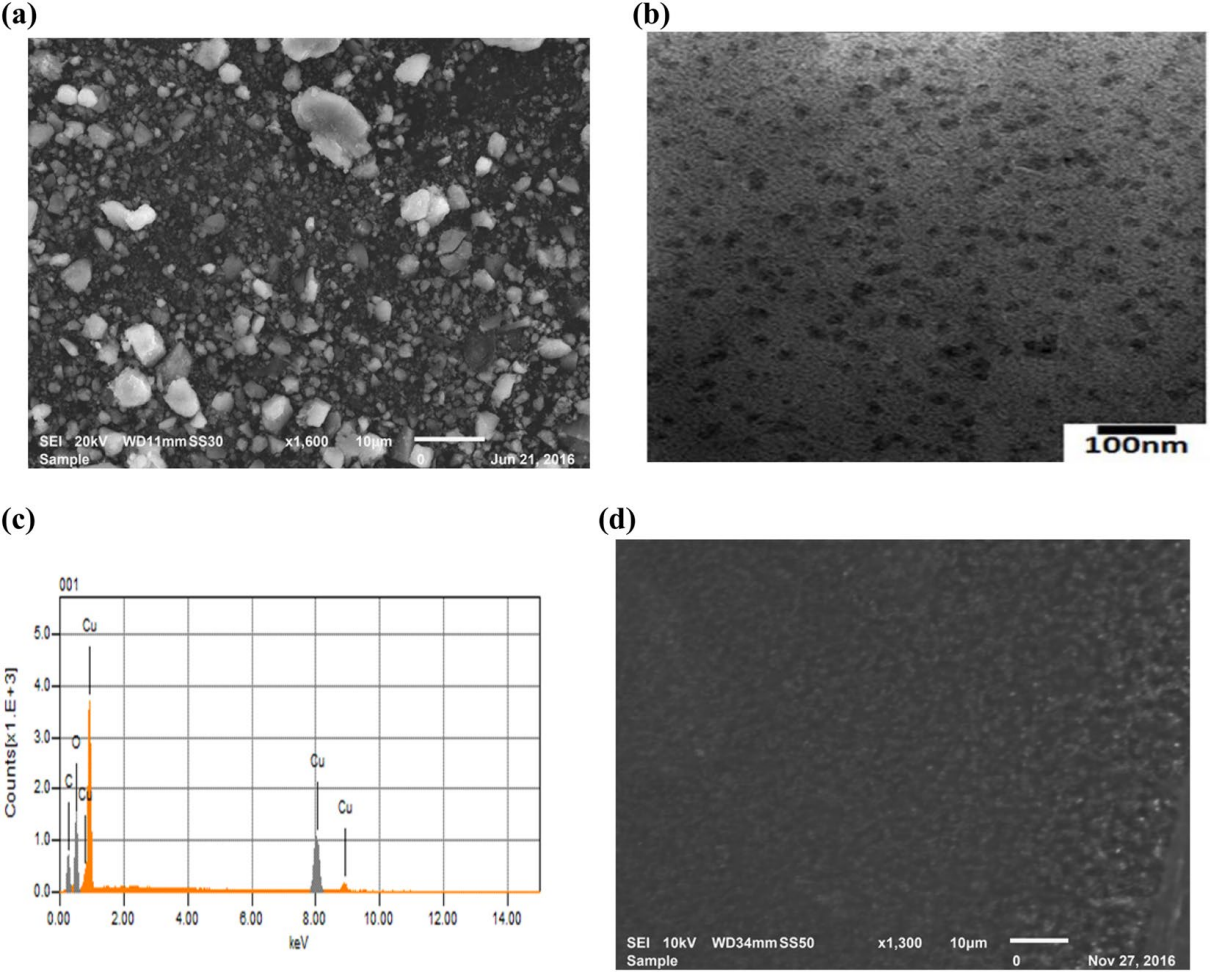
(**a**) SEM image, (**b**) TEM image, (**c**) EDS spectrum of the synthesized CuO NPs, and (**d**) SEM image of the sensing membrane (CMC-IL-5%CuO).

The morphology of the prepared CuO nanoparticles was demonstrated by the SEM and TEM images as shown in Fig. 2(a,b). The figures reveal the agglomerates of CuO nanoparticles. Further analysis with the EDS spectrum (Fig. 2(c)) shows that the formed nanoparticles do not contain impurities. Figure 2(d) presents the SEM image of the (CMC-IL-5%CuO) membrane’s surface, which shows a uniform distribution of the nanoparticles inside the membrane.

**Figure 3:**
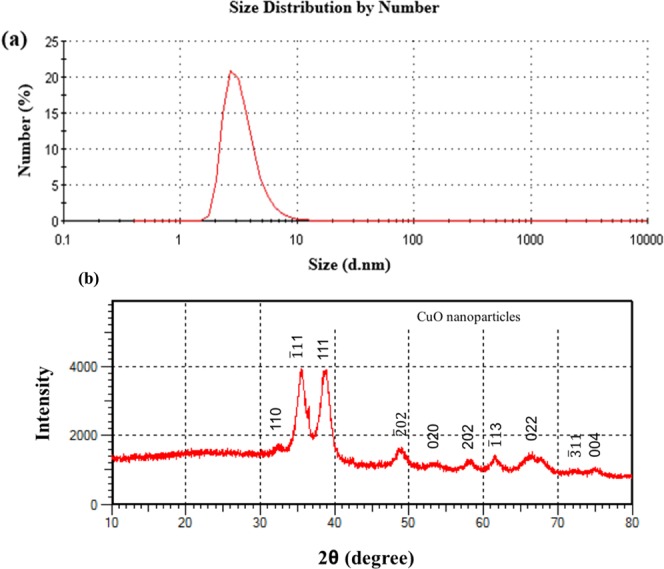
(**a**) Size distribution of the CuO nanoparticles as measured using the Zeta-sizer, and (**b**) XRD spectrum of the CuO nanoparticles.

The size of CuO nanoparticles were evaluated by Zeta-sizer as shown in Fig. 3(a), where the precise particle size was found to be 5.0 ± 1.4 nm. The XRD pattern of the CuO nanoparticles is presented in Fig. 3(b). The XRD peaks of the sample were indexed according to JCPDS card No.83–0950. The peaks well match with the monoclinic crystal structure CuO and confirm the production of CuO nanoparticles. No peaks of impurity are observed in the XRD pattern, indicating the high purity of obtained CuO nanoparticles.

### Electrical characteristics of CMC-CuO composites

**Figure 4:**
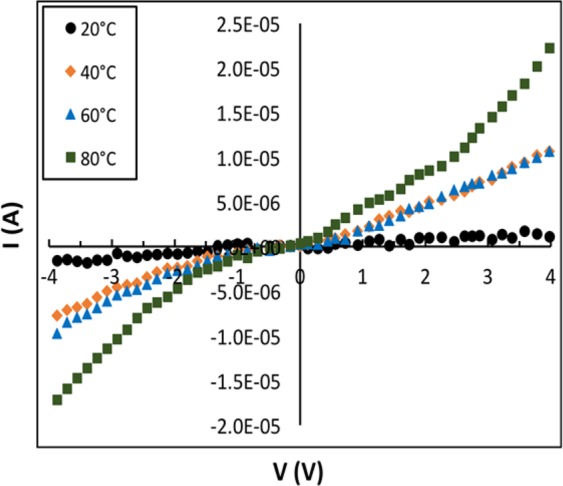
(**a**) I–V characteristics of CMC-IL-5%CuO sensor as a function of temperature.

Before conducting the gas response test, the current-voltage (I–V) measurements were performed. Each sensor device was fixed on the test stage (heating element) inside a Teflon chamber with precise temperature control. The electrical electrodes of the sensor were fixed to the grid and copper contacts by a conductive silver paste. Then, the I–V curves of the sensors were measured at different temperatures. Representative I–V curves of a sensor (CMC-IL-5%CuO) are shown in Fig. 4. The results show an increase in the current (I) as the temperature increases. The temperature-dependence of the current indicates that these sensors are thermally activated, (i.e. they have semiconducting properties). The I–V curves enable selecting the value of the bias voltage to be applied across the sensor during the gas response test, where the electrical current is being measured.

### H_2_S sensor tests

**Figure 5:**
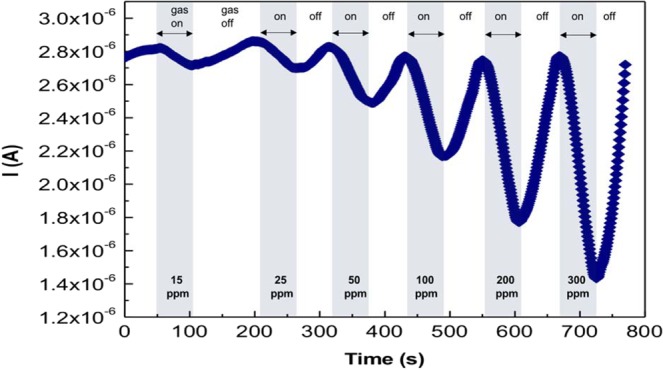
The response curve of the sensors.

The sensing performance of the fabricated sensors was investigated against H_2_S inside a custom-designed Teflon chamber at controlled temperatures. Systematic gas response tests were performed at different gas concentrations and at different temperatures ranges from 40 °C to 80 °C. The sensor response (S%) is defined as:

1 $$S \% =\frac{|{I}_{g}-{I}_{0}|}{{I}_{0}}\times 100$$

where I_g_ is the electric current in the presence of H_2_S gas and I_o_ is the reference electrical current in the absence of H_2_S gas (only air is supplied). During the test, H_2_S gas was added with controlled concentrations, and it was diluted with air inside a gas mixer and injected into the chamber with controlled flow rates. A bias voltage of 1volt was applied between the sensor electrodes, and its electrical current signal was measured with reference to the time at different H_2_S concentrations. Figure 5 shows the response of the (CMC-IL-5%CuO) sample when tested for different H_2_S gas concentrations at 80 °C.

**Figure 6:**
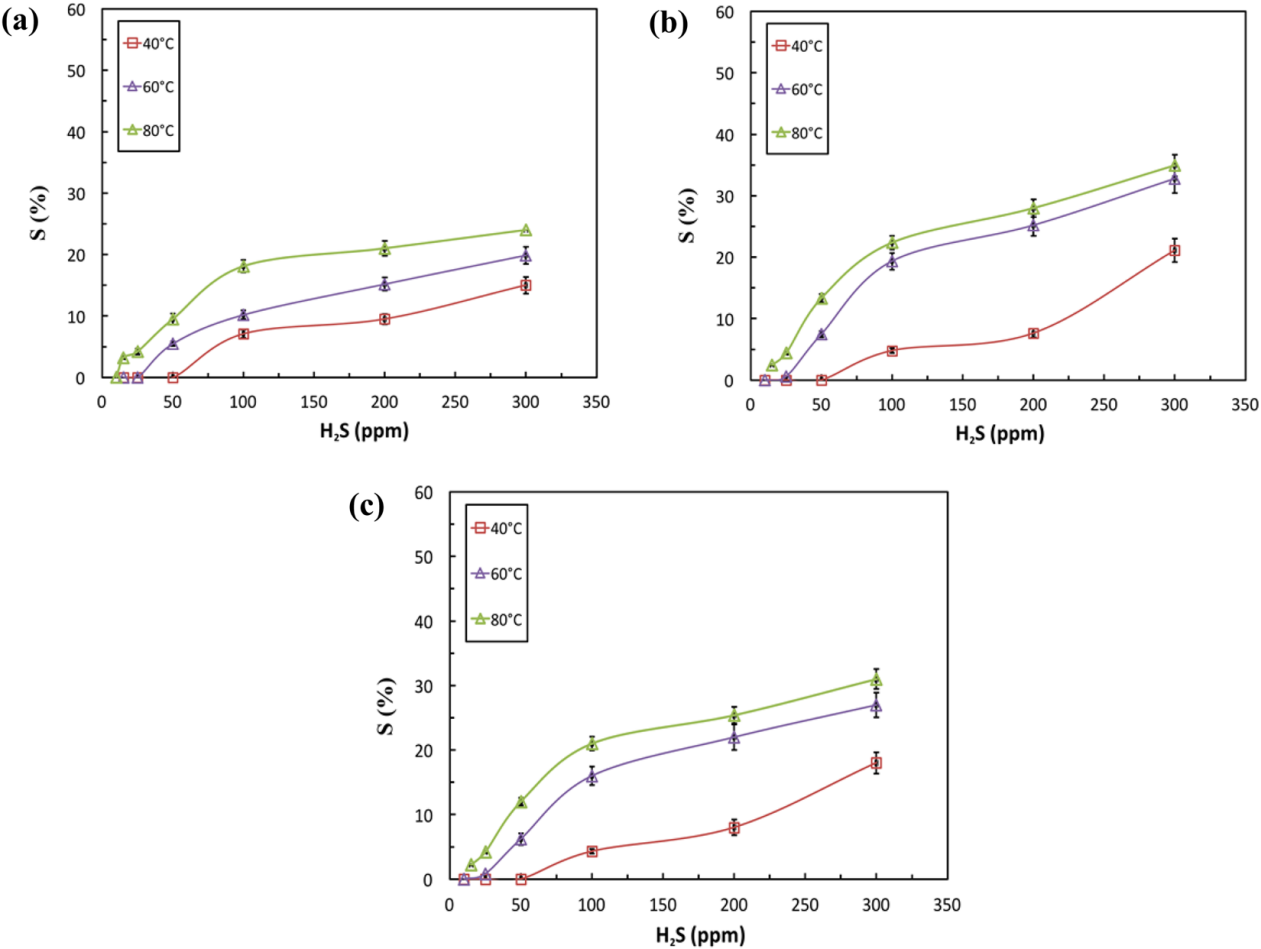
Sensor’s response for H_2_S gas at different temperatures and gas concentrations of: (**a**) CMC-IL-2.5%CuO, (**b**) CMC-IL-5%CuO, and (**c**) CMC-IL-7.5%CuO.

The result clearly reveals an increase in the sensor’s resistance (reduction in electrical current) as the sensor is exposed to H_2_S gas when H_2_S is removed and only air is supplied, the resistance returns back to its initial value indicating a reversible behavior by the sensor. This reversible behavior reflects the ability of the sensor to be used for several times. Moreover, the magnitude of change in resistance is directly proportional to the gas concentration. Figure 6(a–c) shows the sensors’ responses of the three fabricated sensors to H_2_S gas, as measured at working temperatures in the range from 40 °C to 80 °C. The three sensors were fabricated with CuO NPs concentrations of 2.5, 5, and 7.5%; and they showed a reasonable response at 40 °C and at H_2_S concentration of 15 ppm. These responses are proportional to the gas concentrations and temperatures, in other words, as H_2_S gas concentration and temperature increase, the sensor response increases for all three sensors. When the concentration of CuO NPs increases from 2.5 to 5%, the response increases, while no further increase is observed when the concentration increases to 7.5%. This can be explained as follows: when the concentration of CuO NPs increases from 2.5 to 5%, the number of reactive sites increases, thus, the sensing performance increases. However, this is only true to an optimum CuO NPs concentration, where after that, nanoparticles agglomerate causing a reduction in the number of reactive sites, thus, no further increase is observed in the response as the concentration is increased to 7.5%. Overall, these sensors exhibit the best sensing performance at 80 °C.

The low operation temperature is an indication of the low power consumed by the heater for the sensor to operate. Metal-oxide based sensors are known for their high operating temperature above 200 °C^28,35,36^. Therefore, reducing the operating temperature in this work from 200 °C to 40 °C was found to reduce the power consumed by the heater by around 96%. The power reduction was calculated as follows:

2 $${P}_{reduction}=\frac{{P}_{200^\circ C}-{P}_{10^\circ C}}{{P}_{200^\circ C}}\times 100$$

where P_200 °C_ and P_40 °C_ are the power consumed by the heater to heat it up to 200 °C and 40 °C, respectively. P_200 °C_ = 33.75 W and P_40 °C_ = 1.35 W.

The sensing mechanism of these sensors can be explained based on the adsorption and desorption of oxygen molecules. The oxygen adsorbed on the film surface and it ionized to be in the form of (such as O^−^ and O^2−^). In this reaction which shown in Eq. (3), oxygen molecules act as an electron accepter. Thus, the number of holes increased and that in turn increase the current value. In the H_2_S exposure period, the reducing gas (H_2_S) reacts with adsorbed oxygen according to Eq. (4). The extract electrons fill the neutralize holes in the semiconductors, thus decrease the current value^37^:

3 $${O}_{2}^{-}({\rm{gas}})\to {{\rm{O}}}_{2}({\rm{ads}})+{{\rm{e}}}^{-}$$

4 $$2\,{{\rm{H}}}_{2}{\rm{S}}+3\,{{\rm{O}}}_{2}^{-}({\rm{ads}})\to 2\,{{\rm{H}}}_{2}{\rm{O}}+2\,{{\rm{SO}}}_{2}+6{{\rm{e}}}^{-}$$

5 $${{\rm{e}}}^{-}+{{\rm{h}}}^{+}\leftrightarrow {\rm{Null}}$$

**Figure 7:**
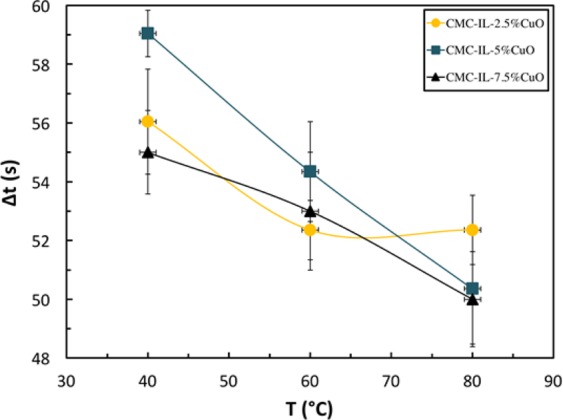
Response times of the sensors as functions of temperature and CuO content.

Hence, during the exposure to H_2_S, the number of released electrons increases according to Eq. (4), which neutralize the holes in the p-type oxide semiconductor (Eq. (5)), thereby increasing the measured resistance^38^. While the ionic liquid and metal-oxide semiconducting NPs initiate paths for the current, the polymer matrix hosts the NPs in the composites. When the flow of H_2_S gas is stopped and only air is supplied to the sensor, the number of free electrons is reduced; thus, the resistance of the sensor almost recovers its initial value indicating reversible behavior. The response time is defined as the time needed for the response of the sensor to reach 90% of its maximum value. The response time of the different sensors is presented in Fig. 7. The error bars are taken as one standard deviation. The minimum average response times are 52.35 ± 3.0 s, 52.40 ± 2.8 s and 50.0 ± 1.6 s for the samples 2.5% CuO, 5% CuO and 7.5% CuO, respectively. These organic-inorganic (CMC-CuO) based sensors are in good agreement with the previously reported sensors for H_2_S gas^38,39^.

**Figure 8:**
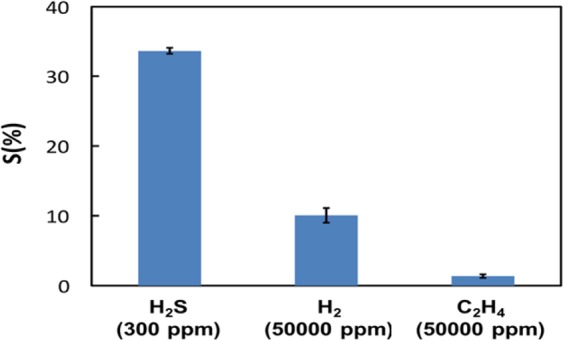
Selectivity test for the CMC-IL-5%CuO sensor for different gases.

Good selectivity is very essential for the optimum operation of a sensor to its target gas. Therefore, in the present work, the response of the developed sensors has been evaluated towards hydrogen (H_2_) and ethylene (C_2_H_4_) gases. Figure 8 shows the corresponding results of the (5%CuO) sensor against H_2_S, C_2_H_4_, and H_2_ gases, measured at 80 °C. The results reveal that the developed sensor has an insignificant response (S%) towards C_2_H_4_ and H_2_ gases compared to its response towards H_2_S gas. As a result, the present sensor exhibits a decent selectivity for H_2_S gas.

**Figure 9:**
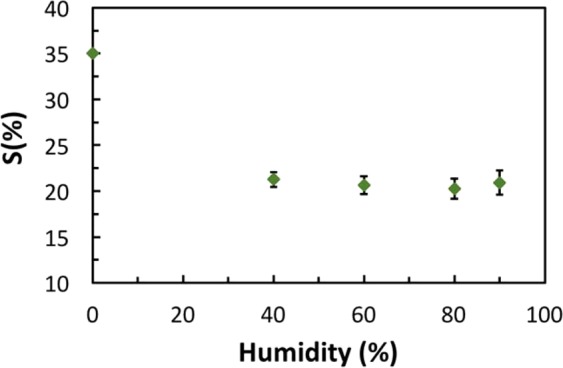
Humidity dependence results of the CMC-IL-5%CuO sensor.

Humidity dependence is a major drawback of some gas sensors due to its negative impact on their responsibilities towards their target gases. Thus, reducing the humidity dependence of the sensor to a low level is desirable. In this work, the humidity dependence of the sensor’s response was tested inside the test chamber at 80 °C and 300 ppm of H_2_S gas for the (5%CuO) sensor, see Fig. 9. The results show a reasonable drop of the sensor’s response from a maximum response of 29% (at dry atmosphere) to around 21% when the relative humidity increases by 40% and above to a very high relative humidity. This result reflects a regenerative interaction between the sensing material and the moisture. Therefore, it indicates the low humidity dependence of the sensor which means good reliability of the sensor that enables its use for practical applications in harsh environments, especially in highly humid atmospheres. This low-humidity dependency result is excellent when compared to some recently reported sensors (Nádherná *et al*.^39^; C. Wang *et al*.^20^).

## Conclusion

In summary, low power consumption and low-cost sensors based on carboxymethyl cellulose (CMC), CuO nanoparticles (NPs) and ionic liquid (IL) were fabricated for H_2_S gas detection. The CuO NPs were synthesized by a colloid microwave-assisted hydrothermal method, and the membranes (sensing materials) were fabricated by the solution casting method. IL was used to enhance the electrical conductivity of the semiconducting membranes. Moreover, the sensors showed noticeable responses at a low temperature (40 °C) with a detection limit of 15 ppm, and a minimum average response time of 52.40 ± 2.8 s. The power consumed by the heating element of this sensor is reduced by 96%. The gas test results showed decent responses and selectivity towards H_2_S gas with relatively short response times and low operating temperatures. In addition, the results revealed low humidity dependence of the sensors. Therefore, these sensors are reliable and they have the potential for practical applications in a harsh environment.

## References

[1] Hwang TY (2019). Rice-like tellurium thin films deposited by a galvanic displacement reaction and ultra-high sensing response to hydrogen sulfide (H2S) gas at room temperature. Sensors and Actuators B: Chemical.

[2] Kim K-H, Choi Y, Jeon E, Sunwoo Y (2005). Characterization of malodorous sulfur compounds in landfill gas,. Atmospheric Environment.

[3] Databank, H. S. Hydrogen sulfide: Environmental Fate & Exposure, *National Library of Medicine, Bethesda, MD*, USA, (2011).

[4] Miyoshi J, Chang EB (2017). The gut microbiota and inflammatory bowel diseases,. Translational Research.

[5] Chou, J. Hazardous gas monitors: a practical guide to selection, operation and applications: McGraw-Hill Professional Publishing, (2000).

[6] Pandey SK, Kim K-H, Tang K-T (2012). A review of sensor-based methods for monitoring hydrogen sulfide,. TrAC Trends in Analytical Chemistry.

[7] McDonagh C, Burke CS, MacCraith BD (2008). Optical chemical sensors,. Chemical reviews.

[8] Safavi A, Haghighi B, Peiravian F (2003). Flow injection analysis of sulfide by gas phase molecular absorption UV/vis spectrometry,. Analytical letters.

[9] Pollard TB, Kenny TD, Vetelino JF, da Cunha MP (2006). Pure SH-SAW propagation, transduction and measurements on KNbO/sub 3,. IEEE transactions on ultrasonics, ferroelectrics, and frequency control.

[10] Wang X, Ding B, Yu J, Wang M, Pan F (2009). A highly sensitive humidity sensor based on a nanofibrous membrane coated quartz crystal microbalance,. Nanotechnology.

[11] Li Z (2015). A fast response & recovery H2S gas sensor based on α-Fe2O3 nanoparticles with ppb level detection limit,. Journal of hazardous materials.

[12] Wu J (2016). Highly selective gas sensing properties of partially inversed spinel zinc ferrite towards H2S,. Sensors and Actuators B: Chemical.

[13] Bakker E, Telting-Diaz M (2002). Electrochemical sensors,. Analytical chemistry.

[14] Fine GF, Cavanagh LM, Afonja A, Binions R (2010). Metal oxide semi-conductor gas sensors in environmental monitoring,. sensors.

[15] Kanan S, El-Kadri O, Abu-Yousef I, Kanan M (2009). Semiconducting metal oxide based sensors for selective gas pollutant detection,. Sensors.

[16] Abu-Hani AF, Awwad F, Greish YE, Ayesh AI, Mahmoud ST (2017). Design, fabrication, and characterization of low-power gas sensors based on organic-inorganic nano-composite,. Organic Electronics.

[17] Sanchez C, Julián B, Belleville P, Popall M (2005). Applications of hybrid organic–inorganic nanocomposites,. Journal of Materials Chemistry.

[18] Koziej D, Fischer F, Kranzlin N, Caseri WR, Niederberger M (2009). Nonaqueous TiO2 nanoparticle synthesis: a versatile basis for the fabrication of self-supporting, transparent, and UV-absorbing composite films,. ACS applied materials & interfaces.

[19] Sabbatini L (1999). Electrosynthesised thin polymer films: the role of XPS in the design of application oriented innovative materials. Journal of Electron Spectroscopy and Related Phenomena.

[20] Wang C, Chu X, Wu M (2006). Detection of H2S down to ppb levels at room temperature using sensors based on ZnO nanorods. Sensors and Actuators B: Chemical.

[21] Ayesh AI, Abu-Hani AF, Mahmoud ST, Haik Y (2016). Selective H2S sensor based on CuO nanoparticles embedded in organic membranes. Sensors and Actuators B: Chemical.

[22] Ali FI, Awwad F, Greish YE, Mahmoud ST (2018). Hydrogen Sulfide (H 2 S) Gas Sensor: A Review. IEEE Sensors Journal.

[23] Josh V, Haik MY, Ayesh AI, Mohsin MA, Haik Y (2013). Electrical properties of sorbitol‐doped poly (vinyl alcohol)–poly (acrylamide‐co‐acrylic acid) polymer membranes. Journal of Applied Polymer Science.

[24] Ayesh AI, Mohsin MA, Haik MY, Haik Y (2012). Investigations on electrical properties of poly (vinyl alcohol) doped with 1-methyl-3-n-decyl-imidazolium bromide ionic liquid. Current Applied Physics.

[25] Allam M, Ayesh AI, Mohsin MA, Haik Y (2013). Physical properties of PVA doped with algal glycerol. Journal of Applied Polymer Science.

[26] Ayesh A, Qadri S, Baboo V, Haik M, Haik Y (2013). Nano-floating gate organic memory devices utilizing Ag–Cu nanoparticles embedded in PVA-PAA-glycerol polymer. Synthetic Metals.

[27] Sothornvit R, Krochta J (2000). Plasticizer effect on oxygen permeability of β-lactoglobulin films. Journal of Agricultural and Food Chemistry.

[28] Hennemann J (2012). Electrospun copper oxide nanofibers for H2S dosimetry. physica status solidi (a).

[29] Das A, Kumar A, Patil NB, Viswanathan C, Ghosh D (2015). Preparation and characterization of silver nanoparticle loaded amorphous hydrogel of carboxymethylcellulose for infected wounds. Carbohydrate polymers.

[30] Basuny M, Ali IO, El-Gawad AA, Bakr MF, Salama TM (2015). A fast green synthesis of Ag nanoparticles in carboxymethyl cellulose (CMC) through UV irradiation technique for antibacterial applications. Journal of Sol-Gel Science and Technology.

[31] Kotresh S (2016). Humidity sensing performance of spin coated polyaniline–carboxymethyl cellulose composite at room temperature. Cellulose.

[32] SwaminathanáIyer K (2012). Pd–sodium carboxymethyl cellulose nanocomposites display a morphology dependent response to hydrogen gas. Green Chemistry.

[33] Ravikiran Y, Kotresh S, Vijayakumari S, Thomas S (2014). Liquid petroleum gas sensing performance of polyaniline-carboxymethyl cellulose composite at room temperature. Current Applied Physics.

[34] Dagher S, Haik Y, Ayesh AI, Tit N (2014). Synthesis and optical properties of colloidal CuO nanoparticles. Journal of Luminescence.

[35] Jundale D (2011). Nanocrystalline CuO thin films for H2S monitoring: microstructural and optoelectronic characterization. Journal of Sensor Technology.

[36] Girija K, Somasundaram K, Topkar A, Vatsa R (2016). Highly selective H2S gas sensor based on Cu-doped ZnO nanocrystalline films deposited by RF magnetron sputtering of powder target. Journal of Alloys and Compounds.

[37] Wang Y (2015). Enhanced H2S sensing characteristics of CuO-NiO core-shell microspheres sensors. Sensors and Actuators B: Chemical.

[38] Mekki A (2014). H2S sensing using *in situ* photo-polymerized polyaniline–silver nanocomposite films on flexible substrates. Organic Electronics.

[39] Nádherná M, Opekar F, Reiter J, Štulík K (2012). A planar, solid-state amperometric sensor for nitrogen dioxide, employing an ionic liquid electrolyte contained in a polymeric matrix. Sensors and Actuators B: Chemical.

